# Marine Biomaterial-Based Bioinks for Generating 3D Printed Tissue Constructs

**DOI:** 10.3390/md16120484

**Published:** 2018-12-04

**Authors:** Xiaowei Zhang, Gyeong Jin Kim, Min Gyeong Kang, Jung Ki Lee, Jeong Wook Seo, Jeong Tae Do, Kwonho Hong, Jae Min Cha, Su Ryon Shin, Hojae Bae

**Affiliations:** 1 Department of Stem Cell and Regenerative Biotechnology, KU Convergence Science and Technology Institute, Konkuk University, Seoul 05029, Korea; zhangxiaowei9304@gmail.com (X.Z.); wjddnr9302@naver.com (J.W.S.); dojt@konkuk.ac.kr (J.T.D.); hongk@konkuk.ac.kr (K.H.); 2 Department of Bioindustrial Technologies, College of Animal Bioscience and Technology, Konkuk University, Seoul 05029, Korea; kkg0118@gmail.com (G.J.K.); vq1004pv@naver.com (M.G.K.); ljk7725@naver.com (J.K.L.); 3 Department of Mechatronics, College of Engineering, Incheon National University, Incheon 22012, Korea; jae.m.cha@gmail.com; 4 Division of Engineering in Medicine, Department of Medicine, Brigham and Women’s Hospital, Harvard Medical School, Cambridge, MA 02139, USA

**Keywords:** fish gelatin, alginate, hydrogel, 3D bioprinting, tissue engineering, bioink, marine products

## Abstract

Biologically active materials from marine sources have been receiving increasing attention as they are free from the transmissible diseases and religious restrictions associated with the use of mammalian resources. Among various other biomaterials from marine sources, alginate and fish gelatin (f-gelatin), with their inherent bioactivity and physicochemical tunability, have been studied extensively and applied in various biomedical fields such as regenerative medicine, tissue engineering, and pharmaceutical products. In this study, by using alginate and f-gelatin’s chemical derivatives, we developed a marine-based interpenetrating polymer network (IPN) hydrogel consisting of alginate and f-gelatin methacryloyl (f-GelMA) networks via physical and chemical crosslinking methods, respectively. We then evaluated their physical properties (mechanical strength, swelling degree, and degradation rate) and cell behavior in hydrogels. Our results showed that the alginate/f-GelMA hydrogel displayed unique physical properties compared to when alginate and f-GelMA were used separately. These properties included high mechanical strength, low swelling and degradation rate, and an increase in cell adhesive ability. Moreover, for the first time, we introduced and optimized the application of alginate/f-GelMA hydrogel in a three-dimensional (3D) bioprinting system with high cell viability, which breaks the restriction of their utilization in tissue engineering applications and suggests that alginate/f-GelMA can be utilized as a novel bioink to broaden the uses of marine products in biomedical fields.

## 1. Introduction

To address the need for cell culture research in native tissue, three-dimensional (3D) cell culture systems have gained increasing attention over the past decade [1,2]. In recent years, hydrogels, which have properties of 3D polymeric networks and a natural extracellular matrix (ECM), have emerged and been widely studied in biomedical fields, including tissue engineering, regenerative medicine, and pharmaceutical processing [3,4]. Both synthetic and naturally sourced hydrogels have facilitated the control of certain physical properties, such as mechanical strength, degradation, swelling, and stiffness, in order to stimulate cells to form functional tissue in a biomimicked physiological state [5]. Due to the lack of biological cues in the case of synthetic hydrogels, bioactive polymers from natural sources have displayed superior biofunctionality over synthetic polymers, as well as low immune response and excellent biocompatibility in biomedical applications [4,5,6,7]. Of the natural resources available, biopolymers from marine sources have been receiving increasing attention as they are free from the transmissible diseases and religious restrictions associated with the use of mammalian resources, as well as the fact that they enable high production at a low cost [7,8,9]. 

In this study, two types of biopolymers, alginate and fish gelatin (f-gelatin), both from marine resources, have been investigated to explore their potential uses in the fields of tissue engineering and 3D bioprinting. Alginate, a polysaccharide derived from brown algae, has been studied extensively and, as a biomaterial, has a broad range of applications in tissue regeneration, tissue repair, and drug delivery [10,11]. For 3D bioprinting applications, alginate has also been used in various printed products due to its fast gelation process via ionic crosslinking and its rheological properties, included in vascular tissue, bone, and cartilage [12,13,14,15,16]. With desirable features like biocompatibility, biodegradability, low cost, and ease of manipulation, gelatin has also attracted increasing interest in the tissue engineering, biomedical, and 3D bioprinting fields [17,18]. However, most of the research in regard to gelatin has focused on mammalian-derived gelatin, and various related factors (such as mammalian diseases and religious restrictions) limit its further development. To address these problems, gelatin from marine resources, such as f-gelatin, provides a promising alternative solution. F-gelatin has been studied extensively and in relation to various applications owing to its significant advantages, low gelling temperature and low melting point. It has a function in pharmaceutical additives, dry products such as nutrition supplements, and microencapsulated vitamins [19,20,21]. However, its weak mechanical modulus and rapid degradation shows that a shortcoming of f-gelatin is its tendency to form stable hydrogel to satisfy the different requirements for various tissue sizes and shapes under biological conditions. Inspired by the gelatin-methacryloyl hydrogels, f-gelatin that is chemically modified by the methacrylation process can be engineered to obtain photocrosslinkable f-gelatin, called f-gelatin methacryloyl (f-GelMA), to address the previously mentioned disadvantages. Our previous work on f-gelatin has showed that photocrosslinkable f-GelMA can be successfully synthesized for tunable physical and biological properties. Moreover, the results demonstrated that f-GelMA can be utilized as a potential substitute for mammalian-derived biomaterials in biomedical applications [7] 

Here, by using alginate and f-GelMA for the first time, we developed an interpenetrating polymer network (IPN) hydrogel and then examined its physical properties and cellular behaviors. Finally, the feasibility of the use of marine based bioink in the 3D bioprinting process was demonstrated in the creation of 3D tissue constructs.

## 2. Results and Discussion

### 2.1. Synthesis of the Alginate/f-GelMA IPN Hydrogel and Mechanical Properties

**Figure 1:**
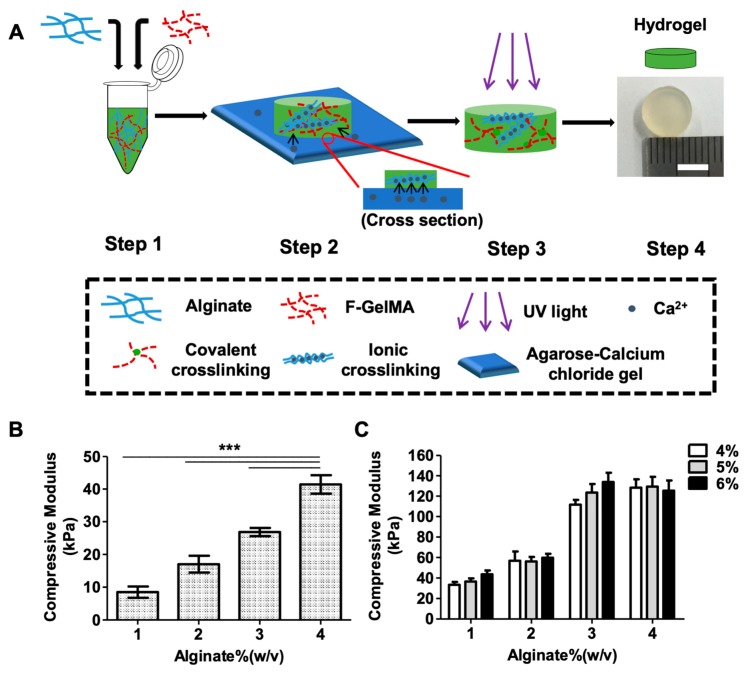
Schematic illustration of fabrication of alginate/f-GelMA hydrogel and mechanical properties. (**A**) Schematic diagram showing the process of fabrication for alginate/f-GelMA hydrogel. Step 1: Hydrogel solution containing alginate, f-GelMA, and 0.5% photoinitiator. Step 2: Polymerization of alginate in the presence of calcium ions (Ca^2+^) diffused from agarose-calcium chloride gel. Step 3: Polymerization of f-GelMA in the presence of photoinitiator under UV illumination. Step 4: photograph of alginate/f-GelMA hydrogel with 8 mm diameter and 2 mm thickness (Scale bar = 5 mm). The composition of alginate/f-GelMA hydrogel was 1%, 2%, 3%, and 4% alginate mixed with 4%, 5%, and 6% f-GelMA, respectively. (**B**) Compressive modulus of different concentrations of pure alginate (*n* = 3 per group and *** *p* < 0.001 was considered statistically significant). (**C**) Compressive modulus of alginate/f-GelMA hydrogels with different compositions.

To optimize the composition of alginate and f-GelMA in bioink and examine its physical properties, alginate/f-GelMA IPN hydrogels with a cylindrical shape, 8 mm diameter, and 2 mm thickness were fabricated, as shown in Figure 1A, and described in the Materials and Methods section. In this study, four different concentrations of alginate (1%, 2%, 3%, and 4%) and three low concentrations of f-GelMA (4%, 5%, and 6%) were investigated and found to form double networked alginate/f-GelMA hydrogels.

**Figure 2:**
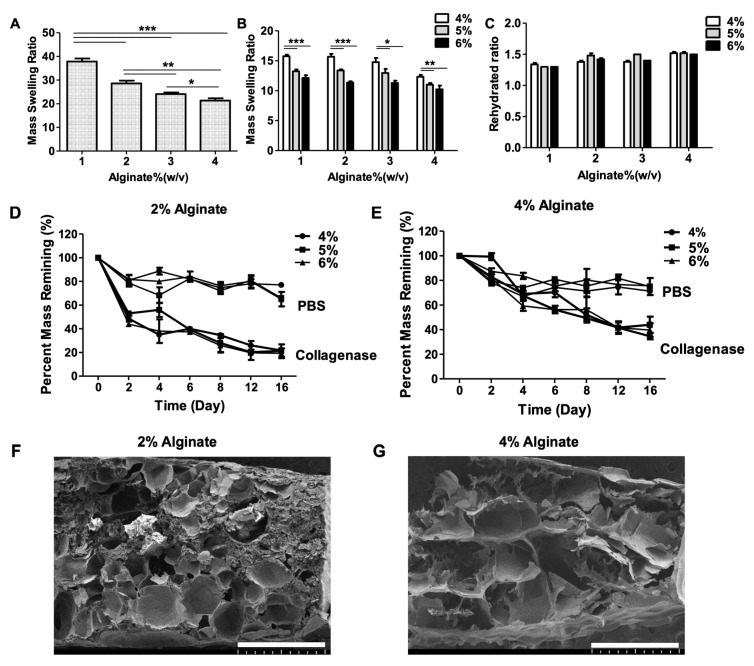
Physical characteristics (swelling ratio, degradation property, and morphology obtained with SEM) of alginate/f-GelMA hydrogels. (**A**) Mass swelling ratios of pure alginate hydrogels at 1%, 2%, 3%, and 4%. (**B**) Mass swelling ratios of alginate/f-GelMA hydrogels consisting of 1%, 2%, 3%, and 4% and 4%, 5%, and 6% f-GelMA. (**C**) Rehydrated ratios of alginate/f-GelMA hydrogels consisting of 1%, 2%, 3%, and 4% and 4%, 5%, and 6% f-GelMA. (* *p* < 0.05, ** *p* < 0.01, *** *p* < 0.001 was considered statistically significant and *n* = 5 per group). (**D**) Degradation property of alginate/f-GelMA hydrogel with 2% alginate and 4%, 5%, and 6% f-GelMA. (**E**) Degradation property of alginate/f-GelMA hydrogel with 4% alginate and 4%, 5%, and 6% f-GelMA (*n* = 3 per group). (**F**) A micrograph obtained using SEM shows a random region of alginate/f-GelMA hydrogel (2% alginate and 6% f-GelMA). (**G**) A SEM micrograph showing a random region of alginate/f-GelMA hydrogel (4% alginate and 6% f-GelMA) (Scale bar = 1 mm).

In the mechanical properties test, the pure alginate hydrogel showed a typical increase in mechanical strength with the increase of concentration and low mechanical strength when its compressive modulus was around 40 kPa, even at 4% alginate, compared with alginate/f-GelMA IPN hydrogel where the modulus of alginate/f-GelMA was approximately 40 kPa at 1% alginate (Figure 1B,C). This phenomenon illustrated that the mechanical strength of hydrogels was significantly increased by employing an alginate and f-GelMA double network. Two facts can be determined from the alginate/f-GelMA mechanical test: 1) In a given concentration of alginate (2% and 4%), there was no difference in the strength when mixed with 4%, 5%, or 6% f-GelMA, but at 1% and 3% alginate, an increase in the mechanical modulus was observed; 2) in a given concentration of f-GelMA, 1% and 2% alginate displayed similar mechanical strength, at low values between 30 and 60 kPa, meanwhile, 3% and 4% alginate displayed high mechanical strength at 110–130 kPa (Figure 2C). Apparently, the tunable mechanical strength range in alginate/f-GelMA hydrogel would be sufficient to meet the diverse requirements of different tissues [22].

### 2.2. Swelling Characteristics

To investigate the swelling behavior we used the aforementioned strategy from the mechanical test to fabricate alginate/f-GelMA hydrogel. We then performed the swelling experiments as described in the Materials and Methods section. With pure alginate hydrogel as a control group, the mass swelling ratio decreased with the increase in concentration of alginate, ranging from around 38 at 1% alginate to 20 at 4% alginate (Figure 2A). For the alginate/f-GelMA hydrogel, the mass swelling ratio for all tested groups was lower than for the pure alginate group (Figure 2A,B). This was due to the increased crosslinking density from the addition of f-GelMA which generated additional polymeric networks via covalent bonding. This behavior was observed in previous studies [23,24]. The 1%, 2%, and 3% alginate/f-GelMA hydrogels showed similar swelling characteristic and the 4% had lower water absorbing capability than the other three groups (Figure 2B). The swelling properties of hydrogel mainly depend on the hydrogel pore size, polymer concentration, density of cross-linking, and the interaction with solvents. Surprisingly, in the rehydrated ratio of hydrogel, all tested groups showed the same trend (Figure 2C). Typically, the rehydration ratio increased with the increasing degree of hydrogel polymerization. However, in this study, the patterns demonstrating the ability of different alginates to restore water were the same after a two-day incubation in Dulbecco’s Phosphate-Buffered Saline (DPBS) [25].

### 2.3. Degradation Characteristics

The stability of hydrogels plays an important role in performance in the cellular microenvironment for tissue engineering and medical applications. For this reason, it is essential to investigate the degradation properties of hydrogels. Alginate hydrogels are not stable in physiological environments in the presence of calcium chelators, whereas f-GelMA hydrogel degradation is an enzyme-mediated process and f-GelMA contains the target sequence of matrix metalloproteinases (MMPs). In the present study, two degradation conditions, Dulbecco’s Phosphate-Buffered Saline (DPBS) and collagenase type II, were used to investigate the degradation properties and experiments were carried out as described in the Materials and Methods section. The 2% and 4% alginate were chosen as the main concentrations for the test. As shown in Figure 2D,E, alginate/f-GelMA hydrogels disintegrated more rapidly with the treatment of collagenase solution than DPBS solution within 16 days. The degradation rate of alginate/f-GelMA in DPBS solution was similar for 2% and 4% alginate, with around 80% hydrogel remaining on day 16. However, 2% alginate/f-GelMA degraded faster than 4% alginate/f-GelMA.

### 2.4. Morphology of Hydrogels

Porosity is an important factor affecting the cell behavior in hydrogels, including cell spreading, supply of nutrients and oxygen, and removal of waste products. As shown in Figure 2F,G, alginate/f-GelMA exhibited a highly porous structure, which can provide enough space for the transport of nutrients and gas exchange for cell survival.

### 2.5. Cell Adhesion on Hydrogel Surface and 3D Cell Encapsulation in Hydrogels

**Figure 3:**
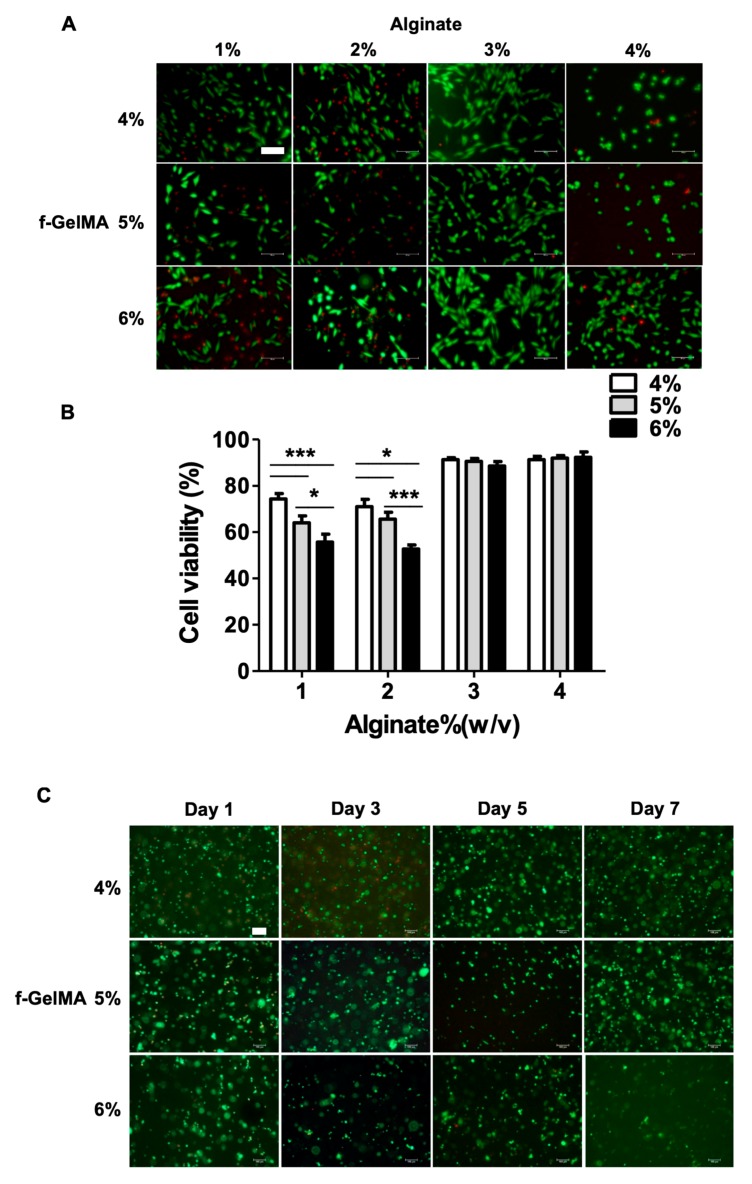
Cell adhesion and 3D cell encapsulation in alginate/f-GelMA hydrogel. (**A**) NIH-3T3 cells in 1%, 2%, 3%, and 4% alginate with 4%, 5%, and 6% f-GelMA (24 h). The live cells are shown in green and the dead cells in red (Scale bar = 100 µm). (**B**) Cell viability on 2D alginate/f-GelMA surface. (* *p* < 0.05, *** *p* < 0.001 was considered statistically significant and *n* = 3 per group) (**C**) NIH-3T3 cell encapsulation in 4% alginate with 4%, 5%, and 6% f-GelMA at day one, three, five, and seven. The live cells are shown in green and the dead cells in red (Scale bar = 100 µm).

To assess the cell behavior and examine the feasibility (cell viability, adhesion, and cell proliferation) of alginate/f-GelMA hydrogel, cell adhesion and 3D cell encapsulation assays were performed to examine the ability to bind to alginate/f-GelMA scaffold which is crucial for cell survival. NIH-3T3 fibroblasts were employed in this study and Live/Dead assay was performed to evaluate cellular viability and visualize cell behavior. NIH-3T3 cells adhered to the surface of alginate/f-GelMA hydrogel with good cell morphology at the concentration of 1%, 2%, and 3% alginate after 24 h of culture (Figure 3A). However, at 4% alginate, the cells still maintained its round shape at 4% and 5% f-GelMA and showed the same cell adhesion as 1%, 2%, and 3% alginate at 6% f-GelMA (Figure 3A). In terms of cell viability, the cell survival ratio decreased with the increasing concentration of f-GelMA at 1% and 2% alginate. At 3% and 4% alginate, no difference was observed between various f-GelMA and high cell viability, over 90%, was observed (Figure 3B). These results illustrated that different ratios of alginate/f-GelMA influenced cell behavior. To further examine the feasibility of alginate/f-GelMA hydrogel for tissue engineering application, 3D cell encapsulation assay was carried out in alginate/f-GelMA hydrogel using NIH-3T3 cells. With high cell viability in cell adhesion assay and mechanical modulus, hydrogels with 4% alginate were chosen for the test as this formulation performed best in the 3D bioprinting process. Encapsulated NIH-3T3 cells were cultured for seven days and cell viability was determined using LIVE/DEAD assay kits. As shown in Figure 3C, cells maintained high viability during the culture period (one, three, five, and seven days) and demonstrated that cells can maintain long-term survival rates in alginate/f-GelMA hydrogel. Because hydrogel stiffness affects cellular morphologies, the higher stiffness in 4% alginate hydrogel may be the potential factor affecting cell elongation in alginate/f-GelMA hydrogels [26].

### 2.6. 3D Bioprinting Using Alginate and f-GelMA Interpenetrating Polymer Network (IPN)

**Figure 4:**
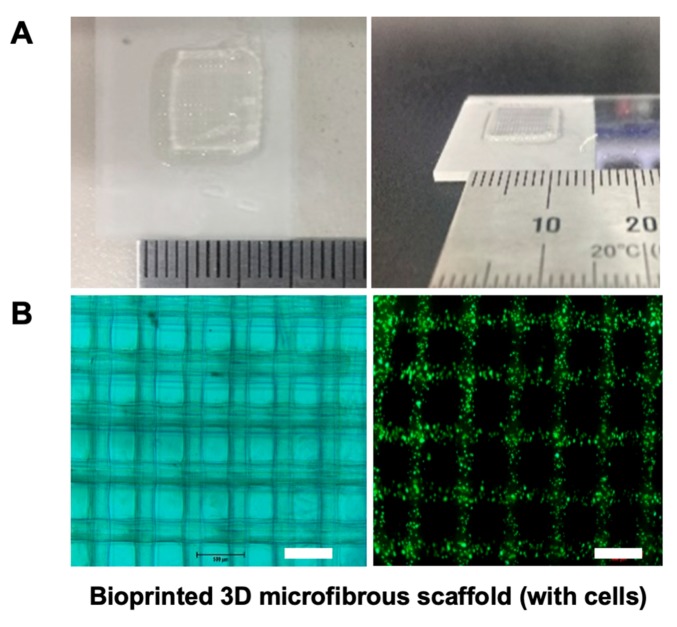

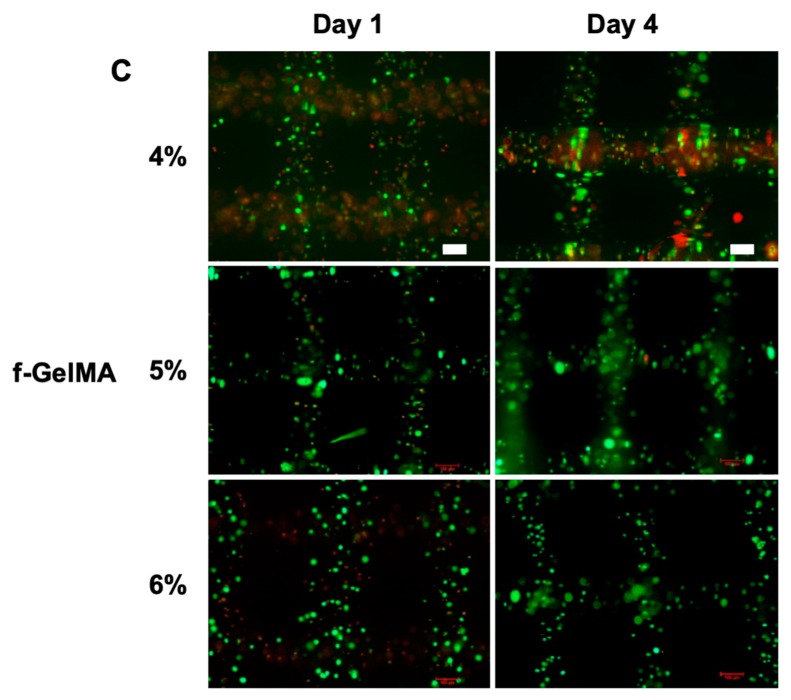
Deposition of cell-laden hydrogel in 3D bioprinting process and cell viability. (**A**) Photograph of printed 3D scaffold. (**B**) Photograph of printed scaffold under microscopy (from left) and Live/Dead cell staining in a two-layer bioprinted scaffold. The live cells are shown in green and the dead cells in red (Scale bar = 500 µm). (**C**) Live/Dead staining with increasing concentration of f-GelMA (4%, 5%, and 6%) in a fixed 4% alginate (Scale bar = 100 µm).

Our previous work showed that f-GelMA can be a promising engineered biomaterial in the tissue engineering and biomedical fields. In this study, to improve the properties of f-GelMA, we first developed an interpenetrating polymer network containing two marine-derived materials, alginate and fish gelatin, to extend the usage of f-GelMA. When examining the physical properties of alginate/f-GelMA, the hydrogels displayed many advantages such as mechanical strength, degradation, long-term survival, and high cell viability. These results showed that alginate and f-GelMA may serve as valuable biomaterials for biomedical applications. One of the most valuable applications of biomaterials is in 3D bioprinting. To examine the feasibility of alginate and f-GelMA polymers for the application of 3D bioprinting, 4% alginate with the highest mechanical strength was selected and subject to testing (with 4%, 5%, and 6% f-GelMA). The cell-laden hydrogel was deposited and the final dimension of the printed product was a 10-layered scaffold which was 10 mm in length and 10 mm in width (Figure 4A). In addition, to further confirm the morphology and the cell viability in the process of printing, a two-layer scaffold was printed and Live/Dead assay was carried out to investigate the cell survival ratio. As shown in Figure 4B, the printer scaffold displayed a satisfactory 3D arrangement under microscopy (left) with high cell viability (right). For the long-term cell survival test (Figure 4C), the composition of 4% alginate and 4% f-GelMA showed lower cell viability than the other two groups and after four days of culture, 4% alginate composed with 5% or 6% f-GelMA maintained a high cell survival ratio.

## 3. Materials and Methods

### 3.1. Materials

Gelatin from cold-water fish skin, methacrylic anhydride (MA), alginic acid sodium salt powder and calcium chloride dehydrate were obtained from Sigma-Aldrich (Sr. Louis, MO, USA). The commercial needles were bought from Korean Good Manufacturing Practices Company (KGMP, Seoul, Korea). Coverslip and microscopy slides were purchased from Marienfeld-Superior (Lauda-König-shofen, Germany). Irgacure photoinitiator 2959 (2-Hydroxy-4’-2(hydroxyethoxy)-2-methylpropiophenone) was obtained from BASF (Ludwigshafen, Germany). The ultraviolet (UV) light source (Omnicure S2000) was purchased from EXFO Photonic Solutions Inc. (Mississauga, ON, Canada).

### 3.2. Schematic Diagram of Bioprinting 3D Microfibrous Scaffolds

**Figure 5:**
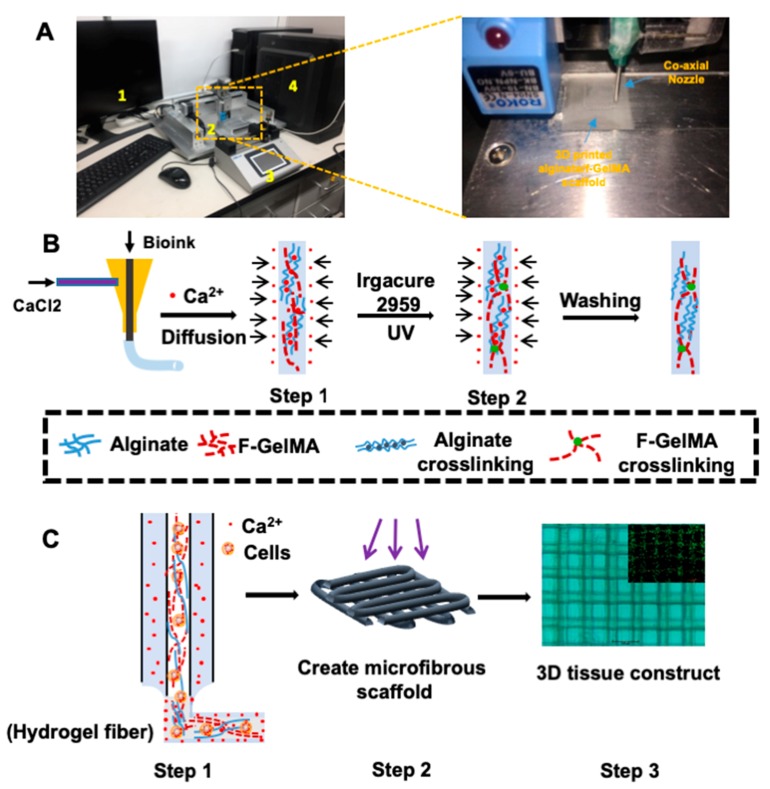
Schematic illustration of 3D bioprinting process using alginate/f-GelMA bioink. (**A**) Left: Photograph of 3D bioprinting system. Right: Photograph of extrusion system of bioprinter. (**B**) Schematic illustration of two-step crosslinking process for hydrogel fibers. Step 1: Ionic crosslinking of alginate in the presence of calcium ion (Ca^2+^). Step 2: Covalent crosslinking of f-GelMA in the presence of photoinitiator under UV light. (**C**) Schematic illustration of the 3D bioprinting process. Step 1: The coaxial system where the bioink was delivered from the core and the ionic crosslinking solution containing calcium ions (Ca^2+^) was on the outer side. Step 2: Schematic diagram to show the designed pattern of hydrogel scaffold. Step 3: Photograph of a bioprinted (two-layer) scaffold with cells (150 µm fiber diameter) and Live/Dead staining image (inset) to show cell viability.

Inspired by applications of f-GelMA in tissue engineering, the current study applied f-gelatin to custom-built bioprinting systems (Figure 5A) by employing bioink, as shown in Figure 5B,C. In order to print f-GelMA, alginate was used to improve the printing ability of f-GelMA as alginate possesses a fast gelation process via ionic crosslinking. In addition, alginate improves the physical properties of f-GelMA hydrogel via IPNs which resulted in the creation of a robust printed construct without any collapse. The 3D bioprinting system that was built consists of four parts (Figure 5A): (1) the computer, (2) the bioprinter (Isolunix, Seoul, Korea) (3) the microfluidic syringe pump, and (4) the connector. In this printing system, commercial needles of different sizes, ranging from 18G to 27G, were used to fabricate the co-coaxial nozzle system for the bioprinting process shown in Figure 5A (left) and Step 1 of the diagram in Figure 5B. Briefly, the 3D printing began as the bioink and calcium chloride solution met at the top of the co-axial nozzle. During the printing process, alginate first undergoes gelation in the presence of calcium ions (Ca^2+^), as shown in Figure 5B,C Step 1, and continued until the microfibrous scaffold was finally printed. Then, the printed scaffolds were exposed under UV light for the desired time until f-GelMA was photocrosslinked to achieve gelation, as shown in Figure 5B,C Step 2. After the washing step, the scaffolds (Figure 5C Step 3) could be employed for further experiments. Meanwhile, to obtain the desired, continuous diameter of printed fiber, the different deposition speeds of the coaxial needle were tested, ranging from 4 to 6 mm/sec, as well as the speed of two flows, ranging from 5 to 10 µL/min. In the final condition, the deposition speed of the coaxial needle at 4 mm/sec and the speed of the two flows at 5 µL/min were employed throughout this study. As shown in Figure 5C, the optimized microfibrous 3D scaffold showed good cell morphology along with high viability (containing 4% alginate and 6% f-GelMA) and verified that f-GelMA could be a promising bioink candidate for 3D bioprinting applications.

### 3.3. Preparation of f-GelMA and Alginate/f-GelMA Hydrogel

F-GelMA was synthesized as described previously [7]. Briefly, gelatin from cold-water fish skin was dissolved in phosphate buffered saline (DPBS; Welgene, Korea) at a ratio of 10% (*w*/*v*). At 50 °C, methacrylic anhydride (MA) was dropped into the gelatin solution to the desired volume (20% of MA) at a rate of 0.5 mL/min and stirred for the reaction. After 2 h, it was diluted 5 times with warm DPBS was added to stop the reaction. The mixture was dialyzed using 12–14 kDa cutoff dialysis tubing for 7 days at 40 °C to exclude impurities. Then, the final dried f-GelMA sample was obtained after lyophilization for 7 to 10 days and stored at 4 °C until further use. The degree of methacrylation for f-GelMA was evaluated using 2,4,6-trinitrobenzene sulfonic acid solution (TNBS) to perform TNBS assay, as described previously.

Alginate/f-GelMA hydrogel was fabricated, as described in Figure 1A, Figure 5B, using a two-step crosslinking [7,27] process. The first step was ionic crosslinking for alginate gelation in the presence of calcium ions (Ca^2+^) that diffused into the hydrogel solution or fiber containing alginate and f-GelMA pre-polymer and replaced the sodium ion for gel formation. The second step was covalent crosslinking for f-GelMA gelation. Through UV irradiation, the f-GelMA pre-polymer underwent chain crosslinking with methacryloyl substitution under the action of the photoinitiator. After the washing step (2–3 times), the hydrogel molds or fibers could be used for further experiments. Three to five 3D scaffold copies were prepared for each group.

### 3.4. Mechanical Test

The Alginate/f-GelMA hydrogel was fabricated as shown in Figure 1A. Lyophilized f-GelMA was fully dissolved in different concentrations of alginate solution containing a 0.5% photoinitiator (Figure 1A Step 1). After removing the bubbles, pre-polymer solution was pipetted into the round shaped polydimethylsiloxane (PDMS) mold (8 mm diameter and 2 mm thickness) and the mold was incubated for 45 min to allow the calcium ions (Ca^2+^) to fully diffuse the whole hydrogel for alginate gelation (Figure 1A Step 2). Then, the samples were exposed to 7.3–7.4 mW/cm^2^ UV light (360–380 nm) for 100 sec (Figure 1A Step 3). After washing (2–3 times with DPBS), the hydrogel samples were incubated in DPBS at 37 °C incubator for 24 h. The following day, the hydrogel discs were tested in compression mode using a CT3 Texture Analyzer (Brookfield Engineering Laboratory, Stoughton, MA, USA) and 5–15% strain was extracted as the slope of the linear region to determine the compressive modulus. Five identical samples were fabricated for each group.

### 3.5. Swelling Test

For swelling analysis, the hydrogels were prepared as described for the mechanical test. The samples were transferred into the 1.5 mL Eppendorf tube and incubated in 1 mL DPBS at 37 °C. After 24 h incubation, the hydrogels were removed from DPBS and washed 2–3 times using distilled water. The swollen weight was recorded after removing excess water. Samples were lyophilized and the mass of dry weight was obtained. The mass swelling ratio was then calculated as the ratio of swollen hydrogel mass to the dried polymer. The mass of the rehydrated hydrogels was weighed after the dried polymers were incubated in DPBS at 37 °C for 48 h. The rehydrated ratio was calculated as the ratio of rehydrated wet mass to the swollen hydrogel mass. The number of samples for analysis was five per group.

### 3.6. Hydrogel Degradation

The hydrogel samples for degradation were fabricated as described for the mechanical test. Hydrogels were transferred in 1.5 mL tubes containing 1 mL DPBS with or without 2 U/mL collagenase type II (Worthington Biochemical Corp, Freehold, NJ, USA). Hydrogels were then incubated at 37 °C for the desired time (2, 4, 6, 8, 12, and 16 days) in the presence of collagenase type II. After each time point, the collagenase solution was removed without disturbing undigested hydrogel. The remaining hydrogel was washed 2–3 times using distilled water and excess distilled water was fully removed prior to lyophilization. The degradation percentage of hydrogel was calculated using the dried weight after digestion divided by the untreated hydrogels. The number of samples for analysis was three per group.

### 3.7. Cell Culture and Cell Characterization

The NIH-3T3 cell line was obtained from the Korean Cell Line Bank (KCBL; Seoul, Korea) and cultured in high glucose Dulbecoo’s modified Eagle’s medium (DMEM; Welgene, Daegu, Korea) containing 10% fetal bovine serum (FBS; Gibco, Waltham, MA, USA) and 1% penicillin/streptomycin (Sigma-Aldrich, WI, USA) at 37 °C. The cells were harvested before performing each experiment.

For cell adhesion studies, circular hydrogels (8 mm diameter and 1 mm thickness) were used, as shown for the mechanical test, and all hydrogels were covered using 3-(trimethoxysilyl) propyl methacrylate (TMSPMA)-coated glass slides during alginate gelation. Then the samples were exposed to 7.3–7.4 mW/cm^2^ UV light (360–380 nm) for 60 sec and washed 2 times using warm culture medium before culturing hydrogels in DMEM in 37 °C incubator. NIH-3T3 fibroblasts were harvested and suspended in the medium at a final cell concentration of 2 × 10^5^ cells/mL. Cell suspension was added to the hydrogel surface and incubated for 24 h in a 37 °C incubator before performing experiments. To evaluate cell viability, NIH-3T3 cells were stained with a Calcein-AM/ethidium homodimer Live/Dead kit (Invitrogen, Carlsbad, CA, USA) after 24 h incubation, according to the manufacturer’s instructions. The images of cells were photographed using a fluorescence microscope (Nikon, ECLIPSE Ts2, Tokyo, Japan) and were analyzed using Image J 1.50a software (NIH, Bethesda, MD, USA).

For 3D cell encapsulation studies, NIH-3T3 cells were harvested and suspended in a hydrogel solution containing 4% alginate, the desired concentration of f-GelMA (4%, 5%, and 6%) and 0.5% photoinitiator at a final cell concentration of 3 × 10^6^ cells/mL. The pre-polymer mixture was pipetted between a TMSPMA-coated glass slide and an agarose-calcium chloride gel coated slide for 1 min. Two microscope slides were separated by a 150 µm spacer. The glass slides containing hydrogels were exposed to 7.3–7.4 mW/cm^2^ UV light (360–380 nm) for 30 sec and washed 2 times before culturing hydrogels in DMEM in a 37 °C incubator. The hydrogels were cultured for the desired time (1, 3, 5, and 7 days) and the medium was changed every 24 h. At each time point, NIH-3T3 cells were stained with a Calcein-AM/ethidium homodimer Live/Dead kit (Invitrogen, Carlsbad, CA, USA) according to the manufacturer’s instructions. The cell images were photographed using a fluorescence microscope (Nikon, ECLIPSE Ts2, Tokyo, Japan) and were analyzed using Image J 1.50a software (NIH, Bethesda, MD, USA).

### 3.8. Morphology of Alginate/f-GelMA Hydrogel

To visualize the morphology of alginate/f-GelMA hydrogel, scanning electron microscope (SEM) images of lyophilized hydrogels were obtained. The alginate/f-GelMA hydrogels were prepared as described in the swelling test. After 24 h incubation, the hydrogels were washed 2–3 times using distilled water and lyophilized. The cross sections of lyophilized hydrogels were exposed and coated with platinum using a sputter coater (Hitachi E-1030, Tokyo, Japan). The images were obtained using SEM (Hitachi Model S-4300, Tokyo, Japan).

### 3.9. 3D Microfluidic Scaffold Printing of Alginate/f-GelMA

The bioprinting procedure using alginate/f-GelMA solution is shown in Figure 5. In the current study, an extrusion system for 3D printing was assembled using two commercially available needles (18G and 27G) in a coaxial configuration system. The 27G needle was inserted into the 18G needle and the needles were attached to each other with epoxy resin (Figure 5C Step 1). Then, the coaxial extruder assembly was mounted on an X-Y-Z moving system using a 3D printer to perform the designed pattern of 3D deposition for hydrogel fibers. The internal needle was linked to 1 mL of plastic tubing containing the bioink (4% *w*/*v* alginate, desired concentration of f-GelMA, 0.5 *w*/*v* photoinitiator, 10% FBS, and 0.25 M 4-(2-hydroxyethyl)-1-piperazineethanesulfonic acid (HEPES), and the external needle was connected with 1 mL of plastic tubing containing the ionic crosslinking solution (0.3 M CaCl_2_, 10% FBS, and 0.25 M HEPES). NIH-3T3 cells at the final concentration of 3 × 10^6^ cells/mL were printed to test the cell viability. The concentration of f-GelMA tested in this work was 4%, 5%, and 6%. A microfluidic syringe pump was employed to control the speed of two flows. For the printing procedure, the deposition speed of the coaxial needle was 4 mm/sec and the speed of two flows was 5 µL/min. Two-layered scaffolds (Figure 5C Step 2) were obtained to perform the Live/Dead staining experiment and the number of scaffolds for analysis was three per group.

### 3.10. Statistics

All data were analyzed using the GraphPad Prism program and are shown as mean ± SD (Standard deviation). Comparisons among the groups were conducted using two-way analysis of variance with Bonferroni post-tests and/or Student’s *t* tests. Statistical significance was considered as a *p* value less than 0.05.

## 4. Conclusions

In this study, the properties of alginate and f-GelMA, both derived from marine source, were determined. To extend the application of fish gelatin for tissue engineering, we successfully generated interpenetrating hydrogels with a double network, which constitutes alginate crosslinking and f-GelMA crosslinking. In the presence of the f-GelMA network, alginate/f-GelMA hydrogel exhibited distinct properties in comparison with the pure alginate and f-GelMA hydrogels, particularly in the areas of mechanical strength, swelling ratio, degradation, and cell behavior. These differences suggest that alginate/f-GelMA may have potential applicability in tissue engineering and satisfy the diverse requirements. More remarkably, it broadens the application of fish gelatin in 3D bioprinting and, considering its high applicability, the use of gelatin derived from fish or other marine products provides an alternative to mammalian gelatin for further exploration in 3D bioprinting.
